# Epidemiology and Outcomes of Pediatric Multidrug-resistant Tuberculosis in Namibia: A Retrospective Review of National Registry Data From 2013 to 2023

**DOI:** 10.1097/INF.0000000000004933

**Published:** 2025-08-06

**Authors:** Ingrid Burkhardt, Nunurai Ruswa, Maria Iitana, Hilya Ekandjo, Mareli M. Claassens, Emmanuel Nepolo, Christoph Aebi, James A. Seddon, Gunar Günther

**Affiliations:** From the * Division of Paediatric Infectious Diseases, Department of Paediatrics, Inselspital, Bern University Hospital, University of Bern, Bern, Switzerland; † National Tuberculosis and Leprosy Programme, Ministry of Health and Social Services, Windhoek, Namibia; ‡ Department of Paediatrics, Katutura Intermediate Hospital, Ministry of Health and Social Services, Windhoek, Namibia; § Department of Human, Biological and Translational Medical Sciences, School of Medicine, University of Namibia, Windhoek, Namibia; ¶ Desmond Tutu TB Centre, Department of Paediatrics and Child Health, Stellenbosch University, Cape Town, South Africa; ‖ Department of Infectious Disease, Imperial College London, London, UK; ** Department of Pulmonology, Allergology and Clinical Immunology, Inselspital, Bern University Hospital, University of Bern, Bern, Switzerland.

**Keywords:** multidrug-resistant, tuberculosis, children

## Abstract

**Background:**

Multidrug-resistant (MDR) and rifampin-resistant (RR) tuberculosis (TB) is challenging the national response to tuberculosis in Namibia. The recent introduction of Xpert MTB/RIF (Cepheid, Sunnyvale, CA) and the use of new and repurposed drugs have the potential to improve both management and outcomes.

**Methods:**

Retrospective review of Namibian national registry data from 2013 to 2023 of children 0–14 years with MDR/RR-TB. National census data were used to estimate annual case notification rates (aCNRs).

**Results:**

Totally 205 episodes were available for analysis. The median age was 4 years [interquartile range (IQR) 1–10]. Ninety (43.9%) were female and 20 (9.8%) were living with HIV. The aCNR increased by two-thirds from 1.2 in 2013 to 2.0 per 100,000 population in 2023. One region, Ojotzondjupa, notified 58 (28.3%) of all cases with a median aCNR of 7.1 per 100,000 population while the national median aCNR was 1.8 per 100,000 population. Ninety individuals (58.1%) received a treatment regimen containing injectables, whereas 65 (41.9.6%) received an all-oral treatment regimen containing two or more World Health Organization class A drugs and/or delamanid. Outcome was unfavorable in 46 (24.1%) individuals, and 18 (9.4%) died. No decrease in the proportion of children with unfavorable outcomes was observed over the study period.

**Conclusion:**

The epidemiology and outcome of children with MDR/RR-TB in Namibia are in keeping with the limited international data available; however, the geographical distribution of children with MDR/RR-TB poses a major challenge to the national TB response.

Estimates suggest that globally 25,000–32,000 children develop multidrug-resistant (MDR; resistant to both rifampin and isoniazid) or rifampin-resistant (RR) tuberculosis (TB) each year.^1,2^ Recent advances in the diagnosis and treatment of MDR/RR-TB, such as the introduction of Xpert MTB/RIF (Cepheid, Sunnyvale, CA) and the use of new and repurposed drugs, influence the management and outcomes of pediatric MDR/RR-TB.

A recent meta-analysis of children and adolescents with MDR/RR-TB found that 72% were successfully treated. Treatment success was positively associated with the use of 2 or 3 World Health Organization (WHO) class A drugs. However, the median age was 16 years, and 89.6% were microbiologically confirmed, with younger and clinically diagnosed children underrepresented. Furthermore, from the WHO African Region, only South African data were available for analysis.^3^

Namibia has a high burden of TB and HIV-associated TB, with an estimated TB incidence of 468/100,000 in 2024 and a HIV co-infection rate of 32%.^4^ Among African countries, Namibia ranks second for the highest population incidence and third for the highest percentage of MDR/RR-TB among individuals newly diagnosed with TB, both markers of community transmission of MDR/RR-TB.^5^ Despite this, there are no published data on pediatric MDR/RR-TB in Namibia, and internal reports from the National TB and Leprosy Program (NTLP) contain limited information on pediatric MDR/RR-TB.

We set out to describe and analyze data on pediatric MDR/RR-TB in Namibia, providing crucial information on the epidemiology and outcomes. In addition to increasing the local understanding of pediatric MDR/RR-TB and informing potential programmatic changes, we hope that this work will generate valuable data to assist other countries in the WHO Africa Region and globally.

## METHODS

### Study Design

This was a retrospective cohort study of children 0–14 years of age registered in the eTB-Manager, the online database of the NTLP of Namibia for drug-resistant TB, between 2013 and 2023.

### Study Setting

Namibia, an upper middle-income country in Southern Africa, has 14 regions and an estimated population of 3 million, of which 37% are children (<15 years).^6^ In 2023, an estimated 14,000 people developed TB, with 1700 of these children (<15 years) and 630 with MDR/RR-TB.^4^ HIV prevalence was estimated at 9.7% in 15–49 year olds in 2023 with a 54% reduction of new infections from 2010 to 2023.^7^ In addition to a reduction in new HIV infections influencing the TB epidemic, substantial changes in diagnosing and managing TB and MDR/RR-TB occurred: Xpert MTB/RIF was introduced as first-line diagnostic test in 2017 and shorter all-oral regimens for MDR-TB were introduced in 2019.^8,9^ Special focus on pediatric TB care was limited due to specialist and economic resource constraints.

### Data Source

Data entered at the district level into eTB-Manager and the publicly available Namibian 2023 Population and Housing Census were used.^6^ The dataset generated is not publicly available; on reasonable request, the corresponding author will forward the request to the Ministry of Health and Social Services Namibia, the custodian of the data, who will decide if the data can be shared.

### Study Population

All children (<15 years) notified in eTB-Manager between January 01, 2013 and December 31, 2023 and a diagnosis of MDR/RR/(pre-) extensively drug-resistant (XDR) TB were included.

### Ethics

Ethics approval was granted by the University of Namibia (Ref. SOM24/2024) and the Ministry of Health and Social Services of Namibia (Ref. 22/3/1/1).

### Definitions

The type of patient was summarized as “new” and “previously treated,” the latter being a combination of “relapse,” “previous failure” and “after default.” HIV status was recorded at diagnosis and monthly during treatment. We defined a child as “HIV-positive” if a positive HIV test was recorded, “HIV-negative” if only negative and “HIV missing” if no results were recorded, respectively. Microscopy and culture results were recorded at the time of diagnosis and monthly during the treatment. We defined each as “positive” if any microscopy or culture was positive, “negative” if all recorded results were negative and “missing” if no result was recorded. Xpert MTB/RIF results were not recorded as such, but after the introduction of Xpert MTB/RIF in 2017, the resistance profile of those children with a positive Xpert MTB/RIF was recorded as RR; thus, after 2017, RR was used as a proxy for microbiologic confirmation by Xpert MTB/RIF. Due to the limited number of recorded additional drug susceptibility testing (DST) results, we only used the variables “DST performed” if any DST results were recorded and “DST not performed” if no result was recorded. Drug resistance categories were grouped into “MDR/RR” and “XDR,” according to the XDR classification used at the time of diagnosis. No individual was classified as pre-XDR. Standard treatment regimens were grouped into: “injectable containing,” “all-oral with 2 drugs from the WHO class A list of drugs and/or delamanid” and “all-oral with 3 drugs from the WHO class A list of drugs and/or delamanid.” Missing regimens were due to missing documentation, no treatment being initiated or individualized regimens being used. Individualized regimens were not reviewed. Outcome variables were divided into “favorable” (cured and treatment completed) and “unfavorable” (failed, lost to follow-up, died and other).

### Data Cleaning

Individuals with multiple baseline data fields (such as age, weight, date of birth and date of registration) that were inconsistent/incompatible with each other were excluded. We further excluded individuals with the outcome “diagnosis changed” and episodes assessed as duplicates. In total, 12 episodes were excluded. If variables describing related characteristics were incompatible (patient classified as new patient but previous treatment episodes documented), they were excluded from the relevant analysis. Individuals with treatment outcome “on treatment” were excluded from the outcome analysis.

### Analysis

The statistical analysis was conducted using STATA Now/BE 18.5, StataCorp LLC. Descriptive statistics were performed. Missing data were excluded except for HIV status and diagnostic tests. Continuous variables were used for age, delay in starting treatment and treatment duration. The Mann-Whitney *U* test was used to assess the effect of age, treatment delay and duration of treatment, given the non-normal data distribution. Risk factors for outcomes were assessed using logistic regression. The notification rate per population was calculated using 2011 and 2023 census data.^6^ Linear population growth was assumed.

## RESULTS

### Epidemiology

**FIGURE 1.**
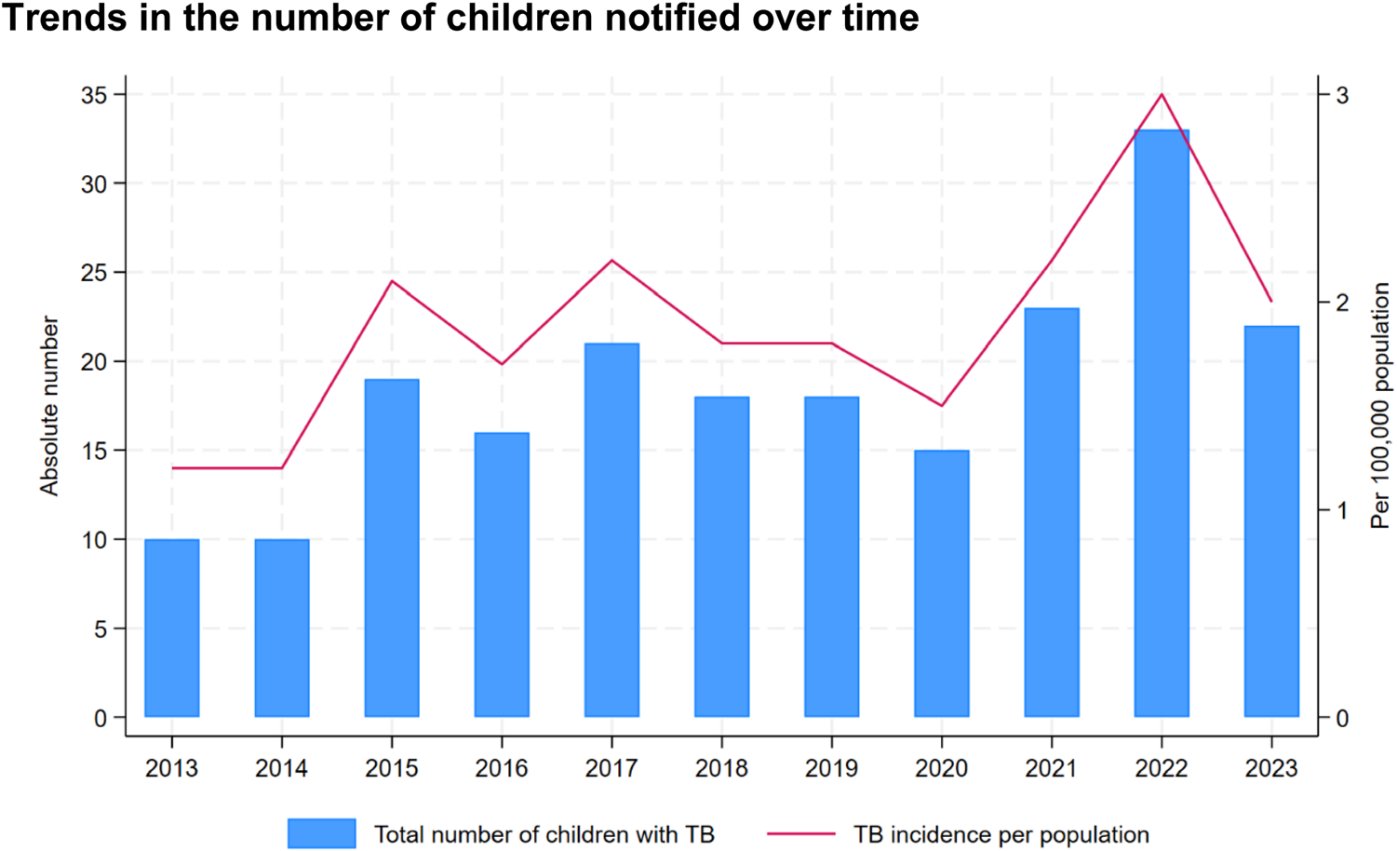
Trends in the number of children notified over time: The absolute number of children notified per year (blue bar, left axis) and the number of notified children per 100,000 population per year (red line, right axis) are shown in this figure. There is a general upwards trend for both. The low of 2020 coincided with the first year of the COVID-19 pandemic. Thereafter, the absolute number and per population incidence peaked in 2022.

After data cleaning and de-duplication, 205 episodes were available for analysis. The annual case notification rate (aCNR) increased by two-thirds from 1.2 in 2013 to 2.0 per 100,000 population in 2023. In 2020, there was a slight dip in notifications coinciding with the first year of the COVID-19 pandemic. Cases peaked in 2022 (Fig. 1).

### Patient and Disease-specific Characteristics

The median age was 4 years [interquartile range (IQR) 1–10]. Ninety (43.9%) were female. Twenty (9.8%) were living with HIV, and 6 (2.9%) had no HIV status documented. Also, 45 (25.1%) individuals had previously had TB (Table 1).

**TABLE 1.** Baseline Characteristics and Stratification by Unfavorable Outcome and Death

Characteristic	Variable	No. (%)	Definitive Outcome Available	Unfavorable Outcome (%)	Died (%)
Total	Episodes	205 (100)	191/205	46 (24.1)	18 (9.4)
Sex	Female	90 (43.9)	83/90	21 (19.4)	11 (13.3)
	Male	115 (56.1)	108/115	25 (30.12)	7 (6.5)
Age (years)	0–1	55 (26.8)	52/55	15 (28.6)	5 (9.3)
	2–4	52 (25.4)	47/52	11 (23.4)	5 (10.6)
	5–9	46 (22.4)	42/46	10 (23.8)	4 (9.5)
	10–14	52 (25.4)	50/52	10 (20.0)	4 (8.0)
HIV status	Positive	20 (9.8)	19/20	5 (26.3)	5 (26.3)
	Negative	179 (87.3)	166/179	38 (22.9)	12 (7.2)
	Missing	6 (2.9)	6/6	3 (50%)	1 (16.7)
Type of patient	New	134 (74.9)	127/134	22 (17.3)	11 (8.7)
	Previously treated	45 (25.1)	40/45	7 (17.5)	4 (10.0)
Site of disease	Pulmonary	153 (83.2)	144/153	28 (19.4)	14 (9.7)
	Extrapulmonary	27 (14.7)	24/27	3 (12.5)	1 (4.2)
	Both	4 (2.2)	3/4	0	0
Culture	Positive	69 (33.7)	68/69	15 (22.1)	6 (8.8)
	Negative	48 (23.4)	46/48	6 (13.0)	2 (4.4)
	Missing	88 (42.9)	77/88	25 (32.5)	10 (13.0)
Microscopy	Positive	59 (28.8)	57/59	13 (22.8)	7 (12.3)
	Negative	85 (41.5)	81/85	16 (19.8)	6 (7.4)
	Missing	61 (29.8)	53/61	17 (32.1)	5 (9.4)
Type of diagnosis	Confirmed	147 (71.7)	138/147	28 (20.3)	14 (10.1)
	Clinical	58 (28.3)	53/58	18 (34.0)	4 (7.6)
DST	Available	50 (24.4)	48/50	11 (22.9)	5 (10.4)
	Missing	155 (75.6)	143/155	35 (24.5)	13 (9.1)
Resistance	MDR/RR	170 (98.8)	158/170	31 (19.6)	17 (10.8)
	Pre-XDR/XDR	2 (1.2)	2/2	1 (50.0)	0
Tx regimen	Containing injectables	90 (58.1)	90/90	16 (17.8)	9 (10.0)
	All-oral 2 group A/Dlm	31 (20.0)	24/31	5 (20.8)	1 (4.2)
	All-oral 3 group A/Dlm	34 (21.9)	30/34	7 (23.3)	3 (10.0)
Year of diagnosis	2013	10 (4.9)	10/10	4 (40.0)	1 (10.0)
	2014	10 (4.9)	12/10	3 (30.0)	3 (30.0)
	2015	19 (9.3)	19/19	0	0
	2016	16 (7.8)	16/16	4 (25.0)	2 (12.5)
	2017	21 (10.2)	21/21	2 (9.5)	0
	2018	18 (8.8)	18/20	6 (33.3)	4 (22.2)
	2019	18 (8.8)	18/18	5 (27.8)	1 (5.6)
	2020	15 (7.3)	15/15	6 (40.0)	2 (13.3)
	2021	23 (11.2)	23/23	5 (21.7)	0
	2022	33 (16.1)	29/33	7 (24.1)	4 (13.8)
	2023	22 (10.7)	12/22	4 (33.3)	1 (8.3)
Region	Erongo	25 (12.2)	22/25	11 (50.0)	1 (4.6)
	Hardap	4 (2.0)	3/4	1 (33.3)	1 (33.3)
	Karas	4 (2.0)	4/4	0	0
	Kavango East	22 (10.7)	22/22	2 (9.1)	1 (4.6)
	Kavango West	5 (2.4)	5/5	1 (20.0)	1 (20.0)
	Khomas	33 (16.1)	32/33	11 (34.4)	4 (12.5)
	Kunene	7 (3.4)	7/7	1 (14.3)	0
	Ohangwena	6 (2.9)	6/6	2 (33.3)	1 (16.7)
	Omaheke	7 (3.4)	7/7	2 (28.6)	2 (28.6)
	Omusati	12 (5.6)	12/12	2 (16.7)	2 (16.7)
	Oshana	5 (2.4)	4/5	1 (25)	1 (25)
	Oshikoto	11 (5.4)	11/11	2 (18.2)	1 (9.1)
	Otjozondjupa	58 (28.3)	50/58	9 (18.0)	3 (6.0)
	Zambezi	6 (2.9)	6/6	1 (16.7)	0

Dlm, delamanid; DST, drug susceptibility testing; Tx, treatment; XDR, extensively drug-resistant.

Baseline patient characteristics, disease specifics, diagnostic methods and treatment regimens are displayed together with the annual and geographic distribution of cases. For each characteristic, the proportion of children with unfavorable outcome and death of those with a definitive outcome available is shown.

### Geographic Distribution

**FIGURE 2.**
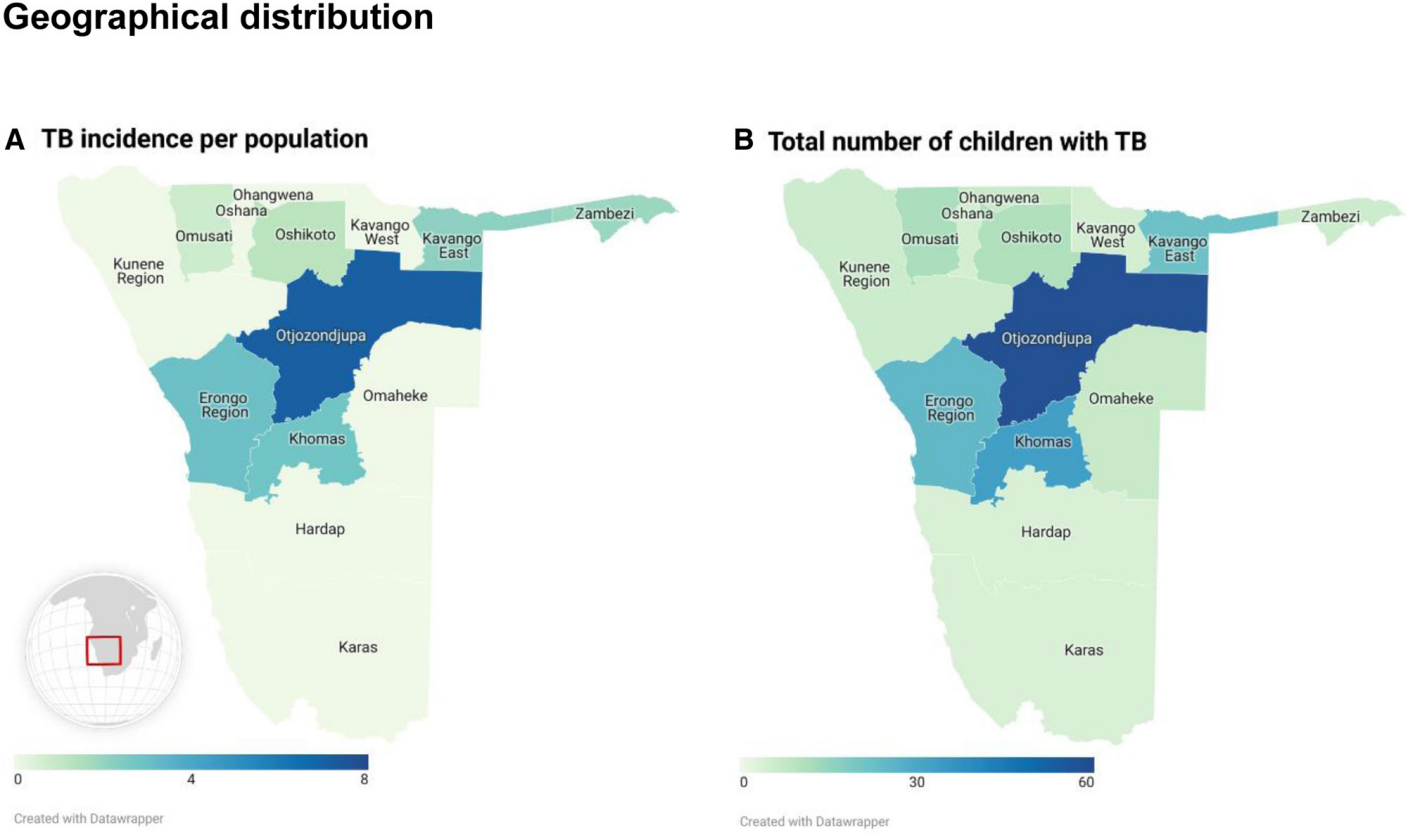
Geographic distribution: Panel A: The median annual number of children notified per 100,000 population (0–14 years of age). Panel B: The absolute number of children notified over the complete study period from 2013 to 2023. Otjozondjupa (dark blue) has the highest rate of cases per population and absolute.

Children were notified from all regions. The total number of individuals ranged from 4 to 58 children per region while the median aCNR per 100,000 population ranged from 0 to 7.1 with a national median of 1.8 (IQR, 1.5–2.2) (Fig. 2). One region, Otjozondjupa, contributed 58 (28.3%) episodes and a median aCNR of 7.1 (IQR, 5.5–8.8) per 100,000 population. Within Otjozondjupa, 54 children (93.1%) were notified through a single health unit serving a rural population in the north-east of the region bordering Botswana, containing the largest settlement of the San population.

### Disease Characteristics

One hundred fifty-three (83.2%) children had pulmonary TB, 27 (14.7%) extrapulmonary TB and 4 (2.2%) had both (Table 1). Of those with known extrapulmonary disease sites, 20 (74.1%) had disease of the lymphatic system, 2 (7.4%) of the spine, 1 (3.7%) of the abdomen and 4 (14.8%) had 2 or more extrapulmonary sites involved. No child with central nervous system (CNS) involvement was recorded. A total of 170 (98.8%) children were classified as having MDR/RR-TB and 2 (1.2%) XDR-TB.

### Diagnostic Methods and Treatment

**FIGURE 3.**
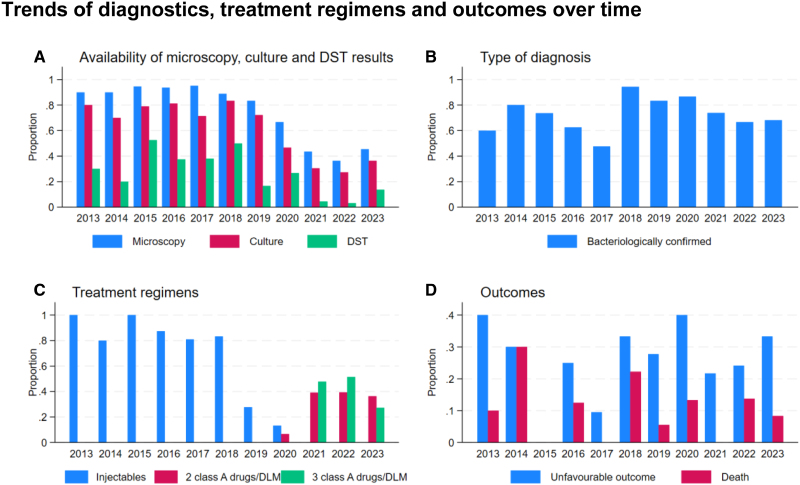
Trends in diagnostics, treatment regimens and outcomes over time. Panel A: The proportion of children with a recorded microscopy result (blue), culture result (red) and DST result (green). Panel B: The proportion of children who had a bacteriologically confirmed diagnosis. The reduction in microscopy and culture performed over time was compensated by the use of Xpert MTB/RIF so that the proportion of bacteriologically confirmed diagnoses remained relatively steady over the study period. Panel C: The standard treatment regimens used over the study period. Up until 2019, the treatment regimen of all children included an injectable (blue). From 2020 onwards, injectable-free regimens containing 2 class A drugs/delamanid (red) or 3 class A drugs/delamanid (green) were being used. Panel D: The proportion of children with unfavorable outcome (blue) and those who died (red). DST, drug susceptibility testing; DLM, delamanid.

Fifty-nine (28.8%) children had a positive microscopy and 69 (33.6%) a positive culture recorded (Table 1). Using the proxy of RR for Xpert MTB/RIF positivity, 78 (38.0%) children were bacteriologically confirmed with Xpert MTB/RIF. Combining microscopy, culture, and RR after 2017, 147 (71.7%) of all children were bacteriologically confirmed. Phenotypic DST results were available in 50 (24.4%) children. The proportion of individuals with recorded microscopy, culture and DST results reduced over time (Fig. 3a) with a reversion of this trend in 2023. Bacteriologic confirmation remained stable over the period (Fig. 3b). The median time from diagnosis to treatment was 6 days (IQR, 1–15), and the median treatment duration was 11 months (IQR, 10–19). The treatment duration significantly decreased from a median duration of 19 months (IQR, 17–23) to 11 months (IQR, 9–13) from 2013–2017 to 2018–2023, respectively (*P* < 0.001). A total of 90 (58.1%) children received a treatment regimen containing injectables, whereas 65 (41.9%) received an all-oral treatment regimen containing 2 or more drugs of the WHO class A drugs category and/or delamanid. Standardized all-oral regimens were introduced in 2019, and the first time a child was registered as being treated with such was in 2020 (Fig. 3c).

### Outcomes

The outcome was unfavorable in 46 (24.1%) children, and 18 (9.4%) children died. Few risk factors assessed were associated with an increased risk for unfavorable outcome (Table 2). There was no association between HIV status, sex, age group, type of patient (previously treated or new) and type of disease and unfavorable outcome. Bacteriologic confirmation was associated with a reduced risk for unfavorable outcome [odds ratio (OR): 0.49; 95% confidence interval (CI): 0.24–1.00; *P* = 0.05]. There was no association between treatment regimens grouped into regimens containing injectables and all-oral regimens containing either 2 or 3 WHO class A drugs and/or delamanid and unfavorable outcome. The median treatment duration was shorter in the group with unfavorable outcome compared with the group with favorable outcome, with 6 months (IQR: 1–10) and 17 months (IQR: 11–20), respectively (*P* < 0.001). The treatment duration was shorter than any of the proposed regimens at the time in those with unfavorable outcomes. The median treatment duration of those children who died was 1 month (IQR: 1–3). No difference in median age and median treatment delay was detected comparing children with favorable and unfavorable outcomes. Over the study period, there was no clear decrease in the proportion of children with unfavorable outcomes or those who died (Fig. 3d). The year of registration was not associated with unfavorable outcomes. Being registered in the regions Kavango East and Otjozondjupa was associated with a reduced risk of unfavorable outcome (OR: 0.1; 95% CI: 0.02–0.53; *P* = 0.007 and OR: 0.2; 95% CI: 0.07–0.66; *P* = 0.007, respectively) compared to the reference of Erongo where 50% had an unfavorable outcome.

**TABLE 2.** Associations Between Baseline Characteristics and Unfavorable Outcome

Characteristic	Variable	OR (95% CI)	*P* value
Sex	Female	1.79 (0.92–3.48)	0.089
	Male	Ref	-
Age	0–1	1.30 (0.51–3.29)	0.58
	2–4	0.98 (0.37–2.60)	0.96
	5–9	Ref	-
	10–14	0.80 (0.30–2.16)	0.66
HIV status	Positive	1.20 (0.41–3.55)	0.74
	Negative	Ref	-
	Missing	3.37 (0.65–17.38)	0.15
Type of patient	New	Ref	-
	Previously treated	1.01 (0.40–2.58)	0.98
Site of disease	Pulmonary	1.69 (0.47–6.07)	0.42
	Extrapulmonary	Ref	-
	Both	1	-
Culture	Positive	1.89 (0.67–5.30)	0.23
	Negative	Ref	-
	Missing	3.21 (1.20–8.55)	**0.02**
Microscopy	Positive	1.20 (0.53–2.74)	0.67
	Negative	Ref	-
	Missing	1.92 (0.87–4.25)	0.11
Type of diagnosis	Bacteriologically confirmed	0.49 (0.24–1.00)	**0.05**
	Clinical	Ref	-
DST	Available	0.92 (0.42–1.99)	0.83
	Missing	Ref	-
Tx regimen	Containing injectables	Ref	-
	All-oral 2 group A/Dlm	1.22 (0.40–3.74)	0.73
	All-oral 3 group A/Dlm	1.41 (0.52–3.84)	0.67

Dlm, delamanid; DST, drug susceptibility testing; Tx, treatment; XDR, extensively drug-resistant.

Odds ratios, 95% CIs and p values for unfavorable outcome are presented. Having no culture performed was significantly associated with unfavorable outcome whilst having a bacteriologically confirmed diagnosis was associated with a reduced risk of unfavorable outcome.

## DISCUSSION

Based on the estimated TB incidence of 1700 (870–2500) children in 2023^4^ and the assumption that about 3% of all children with TB have MDR/RR-TB,^1,2^ the nationwide aCNR suggests that roughly half of the estimated number of children with MDR/RR-TB were notified. The aCNR increased from 1.2 in 2013 to 2.0 in 2023 per 100,000 population, while the estimated incidence (all ages included) of MDR/RR-TB decreased from 36 (21–51) in 2015 to 21 (12–31) per 100,000 population in 2023.^10^ This may reflect improved case finding rather than an increase in numbers. The introduction of Xpert MTB/RIF, active case finding and an emphasis on diagnosing children clinically may have contributed. There was a general upwards trend in case notifications and the low of 2020 coincided with the first year of the COVID-19 pandemic and was observed globally.^4^ It was followed by a compensatory peak in notifications in 2022.

No child had documented CNS involvement. However, a recent mathematical modeling study suggests that the burden of TB meningitis (TBM) is about 2% of the overall estimated TB burden in children under the age of 15 and that about half of those with TBM are being diagnosed and treated.^11^ The observed absence of children with CNS-TB is likely linked to both underreporting and underdiagnosing of this condition.

The proportion of children with bacteriologic confirmation (71.7%), while lower than in other studies^3^ is still higher than the expected 20–30%.^12^ This highlights difficulty and hesitancy to diagnose MDR/RR-TB in children clinically without bacteriologic confirmation and resistance testing in the child, but by using contact history or previous treatment history as indicators for drug resistance.

Children were reported from every region (Fig. 2). Four adjacent regions had the highest absolute number of notifications and aCNRs. Of these, Khomas is where the national capital and the national TB referral center are based, which can, to a degree, explain the high numbers. Otjozondjupa had the highest number of absolute and per population notifications, and most children were notified by a single center, which is not the region’s capital or main referral hospital but is serving a large rural area with limited access to healthcare and transportation services. The Juǀʼhoansi San, an indigenous people of Namibia inhabiting part of Otjozondjupa, are particularly affected by TB.^13^ An active case finding activity in 2021 documented a TB prevalence of 1696/100.000 in one village of the region (personal communication N.Ruswa; NTLP) with 25% MDR/RR-TB. The annual report of the NTLP in 2019 also showed that Otjozondjupa is disproportionately affected by pediatric TB and MDR/RR-TB. In 2021, 17% of all individuals notified were children, whereas nationally, the pediatric proportion was 9.9%.^14^ Our data confirms these regional disparities, which appear to be even more pronounced in children. Otjozondjupa’s median pediatric MDR/RR-TB aCNR is more than 3-fold the national rate and 2-fold that of Khomas. Clustering of MDR/RR-TB strains indicating high levels of transmission has previously been found in Namibia,^15^ supporting our finding suggesting a high level of transmission of MDR/RR-TB in this rural area posing a major challenge to the national MDR/RR-TB response.

A marked reduction in the use of microscopy, culture and DST over the study period was observed. This is likely linked to the introduction of Xpert MTB/RIF in 2017 and repeated stock outs of reagents for culture and DST at the only culture laboratory in the country’s capital (internal communication, Namibia Institute of Pathology Limited, 2023) and potentially the difficulty and reluctance to obtain additional samples for DST once a diagnosis has been made. Diagnosis with Xpert MTB/RIF is an adequate management for children with drug-susceptible TB where no rifampin resistance is detected and suspicion for drug-resistant TB is low. Lack of further DST in patients with confirmed RR-TB risks missing pre-XDR and XDR-TB and other resistance patterns. In addition to low numbers of individuals with XDR-TB in Namibia, the lack of further resistance testing may explain the low number of children notified with pre-XDR-TB and XDR-TB. Given the current drug regimens for MDR/RR-TB, including drugs such as bedaquiline, linezolid and delamanid in shorter all-oral regimens and the globally observed increase in bedaquiline resistance,^16–18^ additional resistance testing is crucial. In 2023, the WHO endorsed targeted next-generation sequencing (tNGS) for the rapid detection of TB drug resistance. Namibia is currently in the roll-out phase with the aim to sequence all MDR/RR-TB strains.^19,20^ This is an exciting advancement with the potential to better understand the extent of circulating resistance other than to rifampin. However, for children, in whom most TB is unconfirmed, tNGS is unlikely to be informative for many.

Favorable outcome was documented in 77.6% of all children, while 9.4% died, both in keeping with a recent meta-analysis, which found favorable outcomes in 80% of children under the age of 15 years, with 9% dying.^3^ Few significant associations between risk factors and outcome were found, which may be partially explained by the small sample size. The association between bacteriologic confirmation and favorable outcome may be because some of the clinically diagnosed children suffered from other undiagnosed and thus untreated conditions. Furthermore, some of the clinically diagnosed children may have had additional resistance and may thus not have been treated adequately. A significantly shorter treatment duration in those children with unfavorable outcomes was found compared to those with favorable outcomes. However, the median treatment durations were shorter than any of the used regimens, indicating that these children were likely sicker when being diagnosed and that unfavorable outcomes occurred either before treatment could be started or completed. The data, therefore, does not indicate that shorter regimens performed worse.

Despite the adoption of Xpert MTB/RIF as the first-line diagnostic test in 2017, with the potential to facilitate the diagnosis of MDR/RR-TB in children and reduce turnaround times, and the introduction of newer all-oral regimens associated with improved outcome,^3^ we saw no reduction in the proportion of children with unfavorable outcome or death over the study period. While between 2020 and 2022 the COVID-19 pandemic may have had a negative impact on outcomes, factors such as access to care, the quality of local services, unsteady drug supplies and comorbidities, such as malnutrition, may have played a major role in limiting the benefits of new diagnostics and treatment regimens and should therefore be investigated in future work.

Our study was limited by small sample size and using routine data, associated with missing data and potentially incorrectly entered data. We attempted to mitigate this by reviewing the data and the exclusion of incompatible data. Data on Xpert MTB/RIF results were not available and only the proxy RR-resistance could be used, limiting the confidence in the categorization of bacteriologically confirmed cases. Furthermore, other than for HIV, data on comorbidities were by default recorded as “not present” and thus not deemed reliable.

Despite these limitations, our study provides important insights into the pediatric MDR/RR-TB epidemic in Namibia and provides a baseline to inform prospective studies as well as programmatic changes which might improve outcomes in children with MDR/RR-TB. It further adds to the sparse data on MDR/RR-TB from the African continent other than South Africa.

## CONCLUSION

The epidemiologic patterns observed in our cohort are mostly reflecting the known pediatric MDR/RR-TB epidemiology and include young and clinically diagnosed children who are underrepresented in other studies. The regional disparities are more pronounced than expected and pose a major challenge to the national TB services. There is a need for further research to better understand local transmission patterns and health system and patient-centered challenges along the whole TB care cascade in areas disproportionately affected by pediatric MDR/RR-TB to inform clinical and programmatic management strategies.
